# Identification of a hippocampal lncRNA-regulating network in cognitive dysfunction caused by chronic cerebral hypoperfusion

**DOI:** 10.18632/aging.103901

**Published:** 2020-10-11

**Authors:** Zhao-Hui Yao, Jing Wang, Bing-Zhen Shen, Yu-Tong Li, Xiao-Li Yao, Shao-Feng Zhang, Yong Zhang, Ji-Chang Hu, Yan-Chun Xie

**Affiliations:** 1 Department of Geriatrics, Renmin Hospital of Wuhan University, Wuhan, China; 2 Department of Pharmacy, Renmin Hospital of Wuhan University, Wuhan, China; 3 Department of Neurology, Central Hospital of Zhengzhou, Zhengzhou, China; 4 Department of Neurology, Renmin Hospital of Wuhan University, Wuhan, China; 5 Department of Pathology, Renmin Hospital of Wuhan University, Wuhan, China

**Keywords:** lncRNA, cognitive dysfunction, chronic cerebral hypoperfusion

## Abstract

Cognitive dysfunction caused by chronic cerebral hypoperfusion is a common underlying cause of many cognition-related neurodegenerative diseases. The mechanisms of cognitive dysfunction caused by CCH are not clear. Long non-coding RNA is involved in synaptic plasticity and cognitive function, but whether lncRNA is involved in cognitive dysfunction caused by CCH has not yet been reported. In the present study, we identified the altered lncRNAs and mRNAs by deep RNA sequencing. A total of 128 mRNAs and 91 lncRNAs were up-regulated, and 108 mRNAs and 98 lncRNAs were down-regulated. Real-time reverse transcription-polymerase chain reaction verified the reliability of the lncRNA and mRNA sequencing. Gene Ontology and KEGG pathway analyses showed that differentially-expressed mRNAs were related to peptide antigen binding, the extracellular space, the monocarboxylic acid transport, and tryptophan metabolism. The co-expression analysis showed that 161 differentially expressed lncRNAs were correlated with DE mRNAs. By predicting the miRNA in which both DE lncRNAs and DE mRNAs bind together, we constructed a competitive endogenous RNA network. In this lncRNAs-miRNAs-mRNAs network, 559 lncRNA-miRNA-mRNA targeted pairs were identified, including 83 lncRNAs, 67 miRNAs, and 108 mRNAs. Through GO and KEGG pathway analysis, we further analyzed and predicted the regulatory function and potential mechanism of ceRNA network regulation. Our results are helpful for understanding the pathogenesis of cognitive dysfunction caused by CCH and provide direction for further research.

## INTRODUCTION

Chronic cerebral hypoperfusion (CCH) exists in many neurodegenerative diseases, such as Alzheimer’s disease [1], vascular dementia [2], and Parkinson’s disease [3], and it functions in the development of these diseases. CCH causes brain tissue to be in a state of hypoperfusion, resulting in ischemia and hypoxia of neural tissue cells, preventing an effective supply of nutrients. Neuronal cells, including neurons and various glial cells in the brain, show oxidative stress [4], calcium overload [5], mitochondrial damage [6], neurotransmitter synthesis dysfunction [7], blood-brain barrier disruption [8], demyelination of nerve fibers [9], and white matter lesions [10]. Furthermore, a variety of signaling pathways are activated in the cell, such as the energy-sensing molecular pathway [11], glucose metabolism pathway [12], kinase pathway [13], cell anabolic [14], apoptotic pathways [15], and so on. These signaling pathways and mechanisms work together in brain cells after CCH, and cause a series of changes in cell function and morphology and aggravation of neurodegeneration based on the existing gene expression changes in neurodegenerative diseases. However, the network relationship between various molecular and pathway changes in the brain tissue after CCH is very complicated, and has not been thoroughly analyzed so far. Therefore, a lack of comprehensive and in-depth understanding of the overall neurological changes caused by CCH remains.

The mammalian genome contains 20,000 protein-coding genes, which represent less than 5% of the genome, and 85% of the genome can be transcribed into RNA, so most of the genome is occupied by non-coding genes [16]. Based on the length of the nucleotides, non-coding RNAs (ncRNAs) of ≥200 nucleotides in length are classified as long ncRNAs (lncRNAs) and those of <200 nucleotides in length are classified as short ncRNAs. LncRNAs are distributed in the nucleus and cytoplasm, as well as in some exosomes and mitochondria [17]. Due to the wide distribution of lncRNAs, their functions are also diverse. Since the introduction of RNA sequencing technology, more and more lncRNAs in different abundances have been identified [18]. This has laid a solid foundation for a comprehensive understanding of the regulatory functions of lncRNA in cells. According to genomic location and structure, lncRNAs can be divided into intergenic lncRNAs away from neighboring genes, anti-sense lncRNAs with sequence elements pairing to other RNAs, sense lncRNAs located within other genes, intronic lncRNAs generated from the introns of other genes, and bi-directional lncRNAs. Among these, intergenic and anti-sense lncRNAs are by far the most common lncRNAs types in mammals [19]. The classifications of lncRNAs can reflect their functions. Anti-sense lncRNAs can bind and regulate their anti-sense genes transcription and act in cis. Trans-acting lncRNAs can also be transported to other locations in the cell to regulate the transcription of genes by acting as scaffolds or decoys to recruit the transcriptional factors, as signaling guides and enhancers of the regulation of mRNA transcription, and by regulating post-transcriptional mRNA levels by sponging miRNAs from binding mRNA to avoid degradation [19–21]. However, the function of many other lncRNAs remains elusive.

Due to the potentially complex and diverse functions of lncRNA, cells contain intricate regulatory networks between lncRNA and mRNA. The lncRNA-regulating network is very susceptible to changes when cells are affected by the external environment, and it is conceivable that they can participate in the regulation of cell functions. LncRNA GAS5 can bind to the PFKFB3 promoter to promote PFKFB3 expression to foster neuronal glycolysis, and this aggravates cerebral ischemia/reperfusion injury [22]. Blocking the binding between lncRNA H19 and miR-19a can improve hypoxia/ischemia-induced neuronal injury [23]. LncRNA-1810034E14Rik has been reported to reduce the expression of inflammatory cytokines and microglia activation in ischemic stroke mice [24]. Previous studies have also shown that many changes in lncRNA expression occur after CCH [25]. After CCH, it remains unclear whether the lncRNA-regulating network in the hippocampus changes, and how it changes. Presently, studies use RNA sequencing technology combined with bioinformatics methods to predict and construct the lncRNA-regulating network of hippocampal tissue after CCH. This facilitates exploration and analysis of the function of lncRNA in cognitive impairment after CCH, and the possible molecular pathways. The analysis of lncRNA-regulating networks is helpful for understanding the key mechanism of the development of cognitive dysfunction and providing a theoretical basis for effective therapeutic targets for CCH.

## RESULTS

### CCH-induced dysfunction of spatial learning and memory in the Morris water maze test

**Figure 1:**
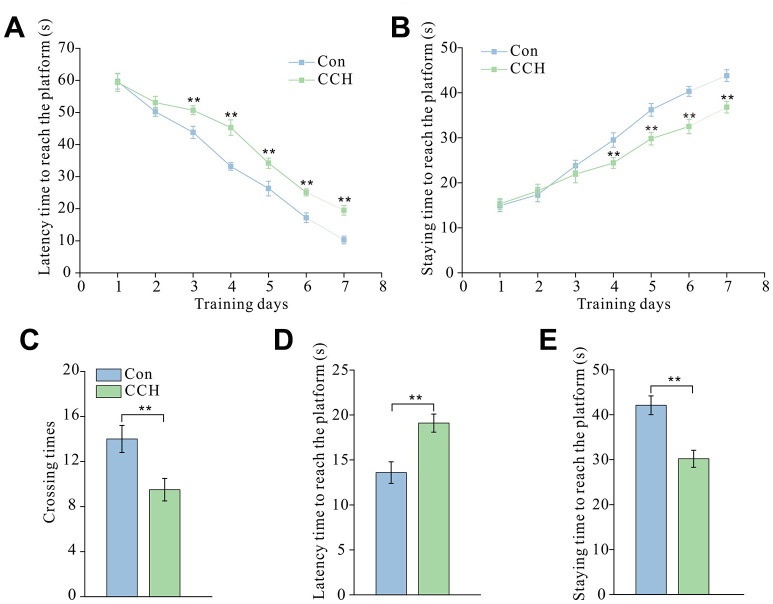
**Chronic cerebral hypoperfusion induced spatial learning and memory impairment in the Morris water maze test.** After a 30-day cerebral hypoperfusion, the rats were trained to learn and remember the location of the platform in the Morris water maze. The latency time to find the platform (**A**) and the staying time in the platform area (**B**) from the first to the seventh day were recorded to evaluate the learning ability of rats. After 1-day rest, the rats were re-tested and the number of times the platform area was crossed (**C**), the latency time to find the platform (**D**), and the time staying in the platform quadrant (**E**) were recorded to evaluate short-term memory. Con: sham group (n=15); CCH: the group with bilateral common carotid artery ligation (n=20). Data are expressed as mean ± SEM. ** *P*<0.01, compared with the Con group.

The results of the Morris water maze test showed that CCH rats had a much longer latency time to reach the platform from the third to seventh training day than the sham control rats (*P* < 0.01) (Figure 1A). CCH rats spent noticeably less time in the platform area from the fourth to seventh training day than the sham control rats (*P* < 0.01) (Figure 1B). After 7 days of training and 1 day of rest, the short-term memory test showed that the CCH rats had a dramatically longer latency time to reach the platform than sham rats (*P* < 0.01) (Figure 1C). After removing the platform, CCH rats spent less time in the platform area and less crossing time around the platform area than the sham control rats (*P* < 0.01) (Figure 1D–1E).

### No obvious number changes of neurons in hippocampus and striatum after chronic cerebral hypoperfusion

**Figure 2:**
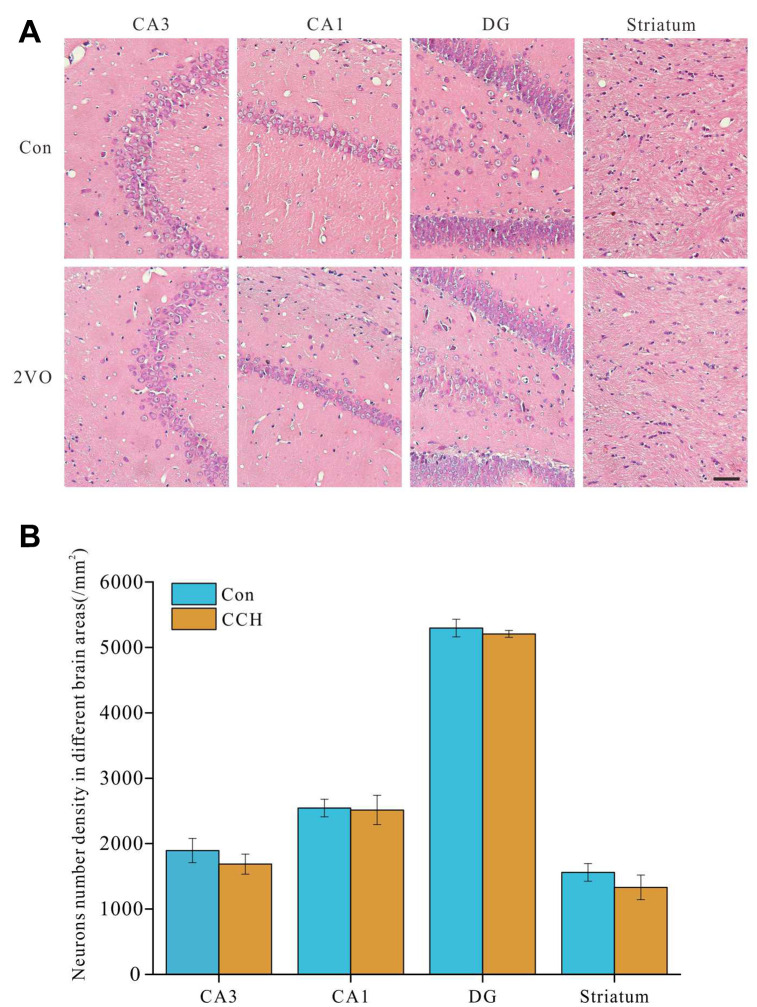
**No obvious number changes of neurons in hippocampus and striatum after chronic cerebral hypoperfusion.** The brain slides were stained with HE staining. (**A**) the images of CA1, CA3, DG of hippocampus and striatum, (**B**) the analysis of the number of neurons in hippocampus and striatum.

In order to explore the potential causes of spatial cognitive dysfunction caused by chronic cerebral hypoperfusion, brain sections were stained by HE staining and the changes of morphology and number of neurons in the sections were observed. The results showed that there was no significant change in the number of neurons in CA3, CA1, DG and striatum (Figure 2A, 2B). This suggested that the cognitive dysfunction caused by chronic cerebral hypoperfusion may not be due to the neurons decrease of in hippocampus and striatum. Scale bar=50μm.

### Expression profile of lncRNA and mRNA profiles in the CCH rat hippocampus

To investigate the underlying mechanisms of cognitive deficit caused by chronic cerebral hypoperfusion, we employed the RNA sequencing to explore the mRNA and lncRNA changes of hippocampus closely related with cognition after chronic cerebral hypoperfusion. Through the assessment of total RNA quantity (≥13μg), the sample concentration (≥433μg/μL), the purity of samples(OD260/280 ≥1.82) and the sample integrity(RIN≥8.5), all extracted RNAs from hippocampus tissue completely meet the requirement of library construct. After high throughput RNA sequencing, 654.12M raw reads and 98.11G raw bases were acquired, containing 340.81M reads and 51.12G for CCH models, and 315.31M reads and 46.99G for the sham controls. Through low quality sequencing data cleaning of the original data, we finally obtained 632.98 M clean reads and 89.54G clean bases in total, containing 329.81M reads and 46.64G bases in the CCH models, and 303.17M reads and 42.9 bases in the sham controls. The proportions of valid bases were 90.75%-91.69% and the Q30s were 94.19~94.68%. The average guanine-cytosine (GC) content was 48.07%.

A total of 22601 protein-encoding transcripts and 15460 lncRNAs were identified and subsequently analyzed in-depth. For lncRNA, 1371 novel lncRNAs were identified and subsequently analyzed. These have not been reported in the past. The sequence length of lncRNA transcripts ranged from 200–4300 bp. A total of 236 mRNAs and 189 lncRNAs were significantly altered in the CCH rat hippocampus compared to sham controls. Among these, 128 mRNAs and 91 lncRNAs were up-regulated, while 108 mRNAs and 98 lncRNAs were down-regulated.

**Figure 3:**
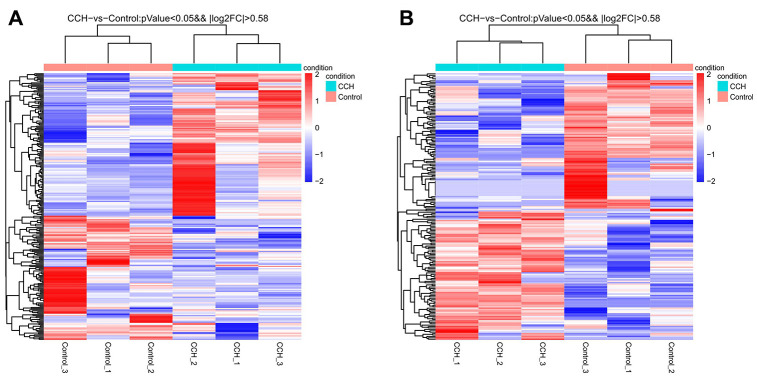
****Heatmap of all differentially expressed mRNA (**A**) and lncRNA (**B**) in hippocampal tissues after chronic cerebral hypoperfusion. Each row represents one miRNA or lncRNA, and each column represents one hippocampal sample. Relative mRNA or lncRNA level is showed by the color scale. Red and blue colors respectively represent high and low relative expression level. The fold changes were normalized and scaled from-2.0 to 2.0 by Z-score.

**Figure 4:**
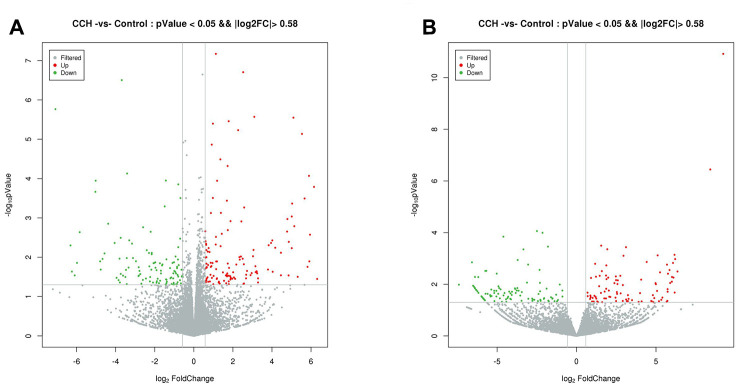
****Volcano plot of differentially expressed mRNAs (**A**) and lncRNAs (**B**). Normalized fold change and P values (CCH group/sham group) were used to construct the volcano plots. The horizontal and vertical lines represents P value and fold change, respectively. The red and green dots represent statistically significantly upregulated and downregulated mRNAs and lncRNAs. The gray dots represent no statistically significantly altered mRNAs and lncRNAs.

The most up-regulated mRNAs and lncRNAs were Sftpa1 and TCONS_00019175, with FCs of 79.54 and 689.55, respectively compared to sham controls. The most down-regulated mRNAs and lncRNAs were Cdh17 and ENSRNOT00000092488, with FCs of 13.38 and 182.18, respectively compared to sham controls. The top 20 up-regulated and 20 down-regulated lncRNAs and mRNAs in the CCH group are listed in Tables 1 and 2. The clustering analysis and volcano plot visualization showed dramatically different expression levels of mRNAs and lncRNAs in the CCH and sham groups (Figures 3 and 4).

**Table 1:** Top 40 differently expressed mRNAs determined by sequencing analysis.

**SeqID**	**P-value**	**Fold change**	**Log2FC**	**Regulation**	**Location**
Sftpa1	0.035649498	79.54236679	6.313651585	Up	Chr16:18716019-18719404
Tmem72	0.000162756	71.14848534	6.152761139	Up	Chr4:148819906-148845199
Sftpc	0.002670317	61.68503716	5.946848674	Up	Chr15:52211538-52214480
Adipoq	0.012731588	60.29333722	5.913926679	Up	Chr11:81330845-81344488
Cldn2	8.53E-05	59.48058263	5.894346874	Up	ChrX:111122552-111133188
Tmem27	0.017575581	56.48254167	5.819733105	Up	ChrX:32118082-32153687
Sostdc1	0.000320698	50.87297918	5.668827678	Up	Chr6:55812820-55816994
LOC100912642	7.31E-06	46.41535627	5.536530287	Up	Chr5:115021650-115066897
Hnf4a	0.03147996	39.83567939	5.315989275	Up	Chr3:159902441-159965003
Kcnj13	0.001624843	35.32490895	5.142613937	Up	Chr9:94486719-94495333
LOC108348080	2.83E-06	34.25942751	5.098429138	Up	Chr5:137670134-137674730
Fam111a	0.000432214	32.77758573	5.03463769	Up	Chr1:229003778-229019532
Zscan10	0.005874147	32.28482174	5.012784156	Up	Chr10:12929471-12939439
Mfrp	0.00092184	32.25845168	5.01160529	Up	Chr8:48437720-48443421
Ttr	0.00406495	29.07587954	4.861750929	Up	Chr8:48437720-48443421
Sftpd	0.028999273	28.83912105	4.84995529	Up	Chr16:18753535-18766100
Kl	0.001069084	27.64599709	4.7889987	Up	Chr12:942974-987206
Slco1a5	0.002227845	27.37727008	4.774906691	Up	Chr4:176445856-176528117
Pon1	0.007692375	22.00198066	4.459561498	Up	Chr4:30249749-30276297
Tbx22	0.02878544	21.9812802	4.458203507	Up	Chr6:78731738-78782542
Cdh17	0.026567758	0.074755887	-3.741668988	Down	Chr5:24595130-24647265
Tsks	0.003213479	0.074640041	-3.743906412	Down	Chr1:89687761-89704629
LOC100911867	0.043812182	0.072443709	-3.786995785	Down	Chr3:4726787-4777044
LOC103690085	0.010844504	0.071646249	-3.802965019	Down	Chr14:81896533-81910812
Adad2	0.038213527	0.069265151	-3.851726507	Down	Chr19: 46924046-46927731
LOC103692396	0.033343448	0.06448369	-3.954921897	Down	Chr5:96118893-96121269
Slc5a8	0.004340917	0.060175138	-4.054688634	Down	Chr7: 20424418-20472528
LOC103689983	0.001406324	0.047911885	-4.383472628	Down	ChrX: 158620026-158658482
Lilrb3l	0.007988447	0.042010311	-4.573112727	Down	Chr1:63074136-63156261
Tspan4	0.011055516	0.039204835	-4.672824615	Down	Chr1:194203708-194227265
Cxcl9	0.020607605	0.036732847	-4.76678546	Down	Chr11:15116823-15122702
Rs1	0.012726917	0.036012269	-4.79536769	Down	ChrX:34647947-34682011
LOC100910575	0.000113293	0.030713747	-5.024971648	Down	Chr4:173113940-173126275
LOC100912599	0.000217208	0.030408465	-5.039383217	Down	Chr1:31263887-31274006
Adam2	0.002304837	0.017410406	-5.843906346	Down	Chr15:39910586-39959136
Gulo	0.013924598	0.01583012	-5.981183981	Down	Chr15:39875033-39901896
Ccr9	0.028961397	0.014583031	-6.099565609	Down	Chr8:122502967-122519814
Rax	0.023198491	0.013213792	-6.241811661	Down	Chr18:57585248-57589682
Krt15	0.005008875	0.012574843	-6.313315842	Down	Chr10:83785803-83790384
Klkb1	1.72E-06	0.00736717	-7.084673798	Down	Chr16:44946907-44974897

**Table 2:** Top 40 differently expressed lncRNAs determined by sequencing analysis.

**SeqID**	**P-value**	**Fold change**	**Log2FC**	**Regulation**	**Location**	**Strand**
TCONS_00019175	2.12E-05	689.5463744	9.429503771	up	Chr20:8484402-8486647	+
XR_001841711.1	0.005003081	43.69311729	5.449334134	up	Chr16:23558712-23573465	+
XR_352416.3	0.008353892	33.90818162	5.083561514	up	Chr3:66403184-66437012	-
XR_592335.2	0.000489082	20.57844907	4.36306235	up	Chr4:173432829-173449403	-
ENSRNOT00000088984	0.020448703	15.91505066	3.992319844	up	Chr5:114204472-114338491	-
TCONS_00034350	0.018563841	15.32730546	3.938032188	up	ChrX:77357719-77428433	+
XR_001840711.1	0.043787387	10.77804191	3.430023196	up	Chr12:47533276-47551299	-
XR_001836753.1	0.028424077	9.493702429	3.246970831	up	Chr2:157099856-157115301	+
ENSRNOT00000082797	0.048125901	9.080827736	3.182823808	up	Chr17:51948845-51981020	+
TCONS_00004253	0.045912724	9.046206998	3.177313009	up	Chr1:279203035-279273961	-
XR_597041.2	0.037152064	7.718909279	2.948397002	up	Chr18:65731592-65738010	+
TCONS_00030722	0.010640434	7.523148724	2.911336611	up	Chr8:4140617-4245359	+
XR_592129.1	0.032146148	7.348211953	2.87739324	up	Chr4:84793194-84793975	+
TCONS_00013454	0.022839865	6.786643837	2.762698302	up	Chr16:56827038-56827916	-
TCONS_00004252	4.76E-05	6.367618035	2.670753798	up	Chr1:279203035-279273882	-
XR_591644.2	0.008110823	6.062764341	2.599975746	up	Chr3:114310470-114336292	+
XR_360324.3	0.035532206	5.962288925	2.575866288	up	Chr16:8466963-8469867	+
XR_001835788.1	0.002961999	5.595768535	2.484336288	up	Chr1:120919146-120955987	-
ENSRNOT00000092197	0.009893472	5.593120976	2.483653535	up	Chr9:47965251-47970908	+
XR_362349.3	0.036902812	5.442656945	2.444311105	up	ChrX:10450197-10458126	+
ENSRNOT00000092488	0.003679888	0.005488992	-7.509242923	down	Chr4:75593043-75708915	+
XR_001838134.1	0.009844005	0.018397383	-5.76435566	down	Chr5:173138920-173141564	+
XR_001841379.1	0.00675928	0.030261756	-5.046360501	down	Chr15:57879139-57883329	-
XR_001836258.1	0.043519547	0.040314994	-4.632539692	down	Chr1:56023467-56038201	+
XR_001839258.1	0.037684229	0.073426768	-3.76755009	down	Chr8:13878950-13896087	+
TCONS_00013871	0.025346422	0.075680135	-3.723941537	down	Chr17:27509254-27521981	+
XR_353865.3	0.002000053	0.07577333	-3.72216604	down	Chr5:21705806-21716612	+
ENSRNOT00000077084	0.014798712	0.08214642	-3.605658491	down	Chr7:142860559-142861925	-
XR_001836260.1	0.029438614	0.090860932	-3.460196088	down	Chr1:56023468-56038201	+
XR_001836263.1	0.012980699	0.09182131	-3.44502718	down	Chr1:56023467-56038201	+
XR_001840969.1	0.001400121	0.09739795	-3.359964789	down	Chr13:109908788-109955148	+
XR_001836996.1	0.000246091	0.121155025	-3.045073857	down	Chr2:262810481-262873478	-
XR_001835966.1	0.008604154	0.142374068	-2.812241699	down	Chr1:194072468-194078064	-
TCONS_00026111	0.013013726	0.143542523	-2.800449913	down	Chr5:129088651-129297455	-
XR_001838453.1	0.038538215	0.148548241	-2.750996569	down	Chr6:92760107-92773394	+
XR_590384.2	0.000136273	0.177859013	-2.491194014	down	Chr1:157561774-157573264	-
XR_597173.2	0.035083573	0.189302667	-2.401233357	down	Chr19:22220940-22244737	+
XR_001842379.1	0.000279854	0.19964672	-2.324478724	down	Chr19:26267879-26269062	+
ENSRNOT00000089868	0.005257745	0.202684724	-2.302690734	down	Chr19:36242337-36257062	-
XR_352417.3	0.049679474	0.206762346	-2.273954618	down	Chr3:66403184-66417915	-

### Expression profile validation

**Figure 5:**
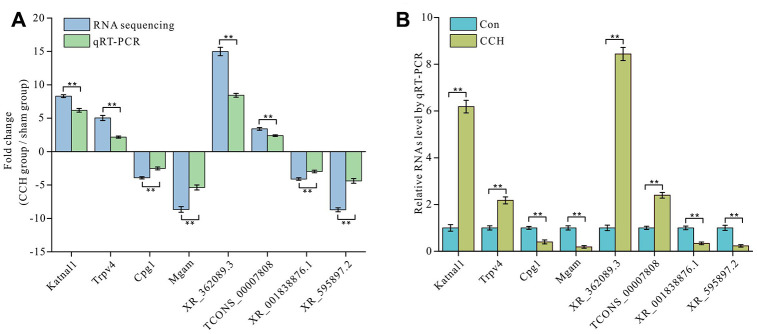
**Validation of mRNA and lncRNA expression level by qRT-PCR.** Two upregulated and two downregulated mRNAs and lncRNA expressions were determined by RNA seguencing and qRT-PCR (**A**) and the detailed validation by qRT-PCR was showed (**B**). The There are three parallel samples to be assayed (n=3). **, P<0.01.

In order to verify the validity of RNA sequencing, we randomly selected two differentially up-regulated mRNAs of Katnal1 and Trpv4 and down-regulated mRNAs of Mgam and Cpg1 and two differentially up-regulated lncRNAs of XR_362089.3 and TCONS_00007808 and down-regulated lncRNAs of XR_001838876.1 and XR_595897.2 for detection by qRT-PCR. The qRT-PCR results showed that the change trends of the selected mRNA and lncRNA levels determined by qRT-PCR were consistent with these by RNA sequencing. This showed that the RNA sequencing data was valid (Figure 5A). The levels of Katnal1 and Trpv4 in hippocampus of CCH group were significantly up-regulated than in that of sham group (*P*<0.01), whereas Mgam and Cpg1 in hippocampus of CCH group were significantly down-regulated than in that of sham group (*P*<0.01) (Figure 5B). Similarly, up-regulation lncRNAs of XR_362089.3 and TCONS_00007808 and down-regulation lncRNAs of XR_001838876.1 and XR_595897.2 were noticeable in CCH group than in sham group (*P*<0.01) (Figure 5B). The primers used in qRT-PCR are listed in Table 3.

**Table 3:** Primers designed for qRT-PCR validation of lncRNAs and mRNAs.

**RNAs**	**Forward primer**	**Reverse Primer**	**Product length (bp)**
Katnal1	5' TGTTGTTTGAAATGGCGAGGTT 3'	5' TCCGTCCATCTGGATGAGGA 3'	143
Trpv4	5' CAAGTGGCGTAAGTTCGG 3'	5' TGCCCTCCAGTGGCTGAT 3'	107
Cpg1	5' TCTCCCAGTGTCGTCGTG 3'	5' TCTTGCCTTTGCGTACA 3'	89
Mgam	5' GCAAGGAGGAAGCGAAAG 3'	5' AGTCCCGTCTCATAGTCA 3'	61
XR_362089.3	5' TGCTGCGACCCTTTGATA 3'	5' AACCTCTAGCGCCGTAT 3'	137
TCONS_00007808	5' CAACCCACTCCAGTCGTCT 3'	5' CAAATCCCAAGGGTCTCCGTTCA 3'	132
XR_001838876.1	5' CTTGAAGGCTGAGGCAGGAGGTT 3'	5' AGGTAGGGTGAGGTAGAATGA 3'	96
XR_595897.2	5' CGAAGCCGTCACAGTGTCTCC 3'	5' TTCAGTCACTCCTGTCATAGCG 3'	132

### GO function and KEGG pathway enrichment analyses of DE mRNAs

**Figure 6:**
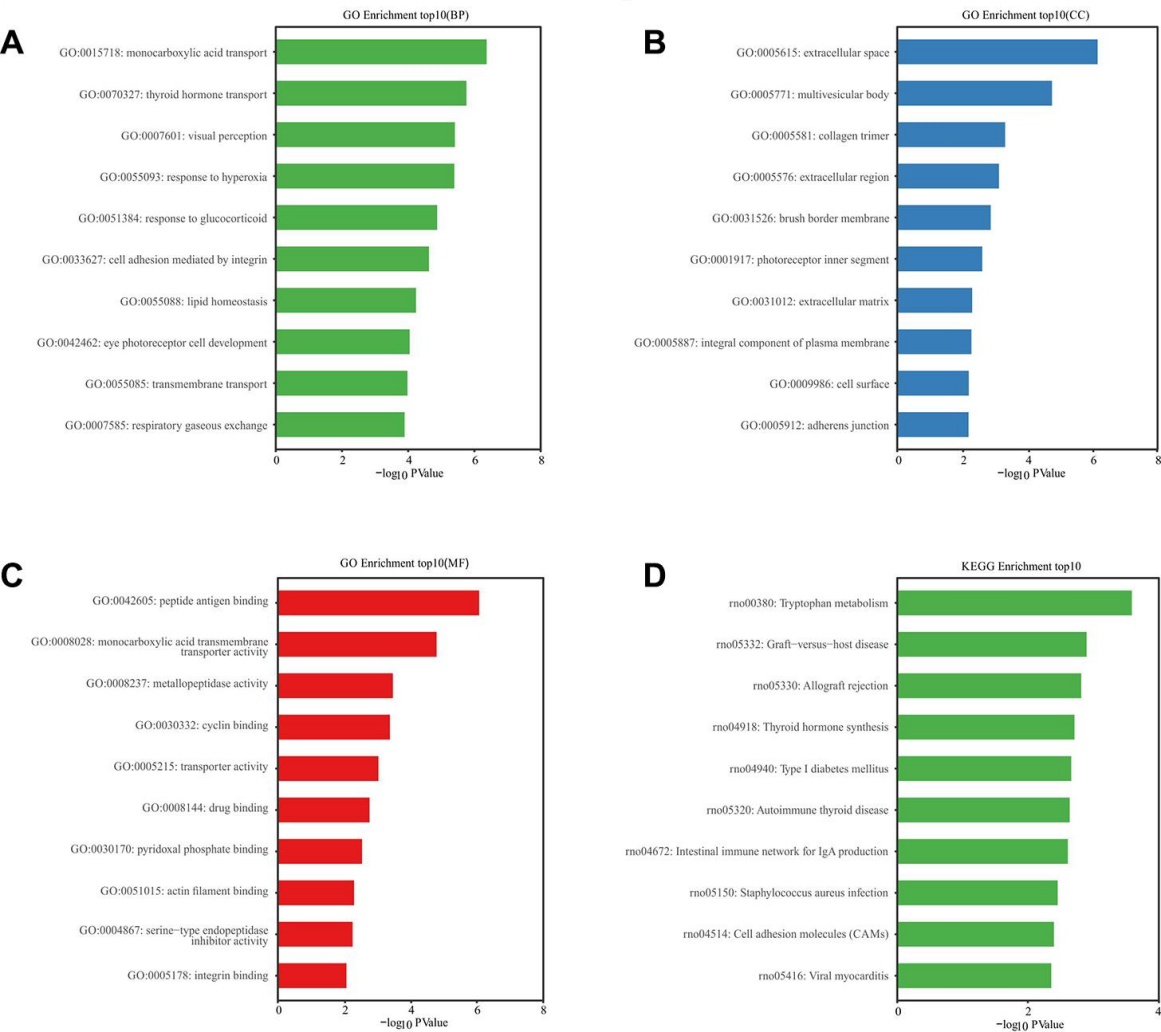
**GO and KEGG pathway enrichment analysis of differentially expressed mRNAs.** P value <0.05 was as significantly differentially enrichment. Enrichment value (-log10 (*P* value)) was calculated and visualized to show the enrichment of biological processes (**A**) the enrichment of cellular components (**B**) the enrichment of molecular functions (**C**) and the enrichment of the KEGG functional pathways (**D**).

The GO analysis predicted, and the KEGG analysis showed, that DE RNAs were associated with the probable functions and pathways. GO analysis indicated that the most enriched mRNAs related to monocarboxylic acid transport (GO: 0015718) in biological processes (Figure 6A), the extracellular space (GO: 0005615) in the cellular component (Figure 6B), and the peptide antigen binding (GO: 0042605) in the molecular functions (Figure 6C). KEGG pathway analysis showed that the top 10 deferentially-enriched KEGG pathways related to dysregulated mRNAs were involved in tryptophan metabolism, graft-versus-host disease, allograft rejection, thyroid hormone synthesis, type I diabetes mellitus, autoimmune thyroid disease, the intestinal immune network for IgA production, staphylococcus aureus infection, cell adhesion molecules (CAMs), and viral myocarditis (Figure 6D).

### LncRNA-mRNA co-expression analyses

**Figure 7:**
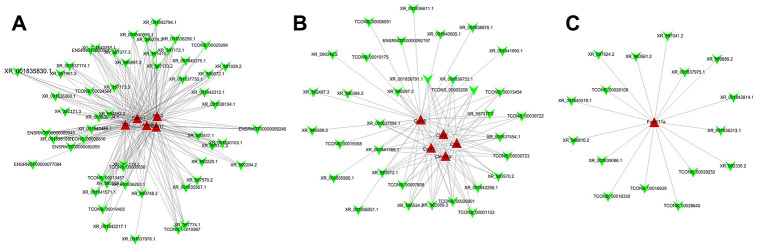
**Co-expression network analysis of differentially expressed lncRNA-mRNA.** After Pearson correlation analysis between differentially expressed lncRNA and mRNA, (**A**) 5, (**B**) 5, and (**C**) 1 mRNA and their corresponding lncRNA with correlation coefficient >0.8 and P value <0.05 were selected to construct co-expressed network of differentially expressed lncRNA and mRNA.

In total, 161 DE lncRNAs that were correlated with DE mRNAs were identified (*P* < 0.05 and correlation coefficient (COR) > 0.8). Among of all the lncRNA-mRNA coexpressions, lncRNA XR_589889.2 and Tgm2 showed *p* = 0.0083 and COR = 0.92 and lncRNA XR_595556.2 and Cdhr3 showed *P*= 0.018 and COR = 0.89. Ten mRNAs and their coexpressed lncRNAs were selected to construct the expression network (Figure 7A–7C).

### Construction of lncRNA-miRNA-mRNA regulatory network

**Figure 8:**
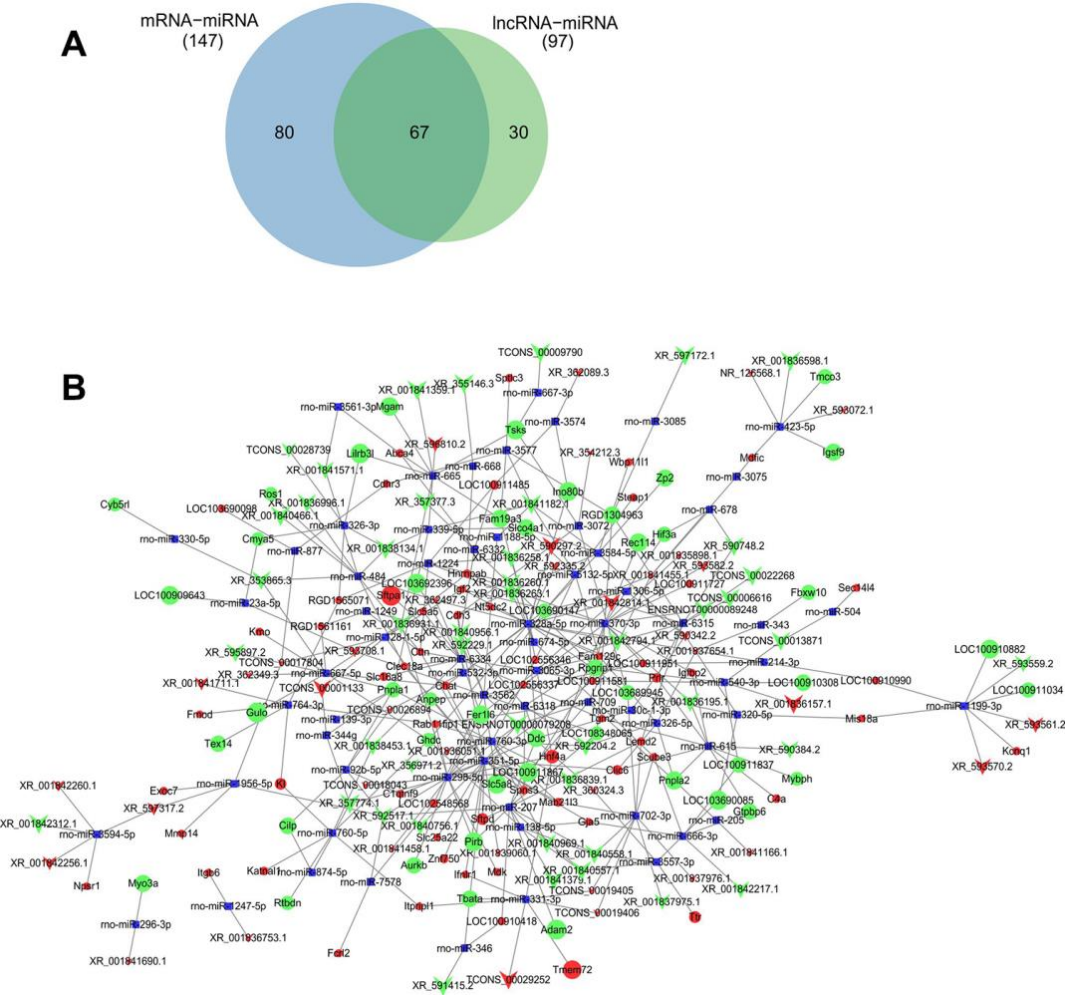
**ceRNAs regulatory network of lncRNA-miRNA-mRNA.** Predicted common miRNA regulated by differentially expressed lncRNA and mRNA (**A**). ceRNAs regulatory network of lncRNA-miRNA-mRNA was constructed with differentially expressed lncRNAs and their predicted binding miRNAs, and differentially expressed mRNA and their predicted binding miRNAs by Cytoscape V3.7.0 software (**B**). Red and green balls represent the upregulated and downregulated mRNAs, red and green arrows represent the upregulated and downregulated lncRNAs, and small blue squares represent miRNA. The size of balls, arrows and squares represents degree of *P* values.

186 lncRNA-miRNA regulatory pairs were identified including 108 lncRNAs and mRNA. 368 miRNA-mRNA target pairs were identified including 147 miRNAs and 130 mRNAs. The 67 common miRNAs were identified (Figure 8A). Because of competitively binding miRNA as a miRNA sponge, lncRNAs could form a ceRNA network of lncRNA-miRNA-mRNA to booster miRNA target genes. Based on the regulatory pairs of miRNA-mRNA and lncRNA-miRNA, an lncRNA-miRNA-mRNA network was constructed. A total of 559 lncRNA-miRNA-mRNA target pairs were identified, including 83 lncRNAs, 67 miRNAs, and 108 mRNAs (Figure 8B).

### Validation of lncRNA-miRNA-mRNA regulatory network

**Figure 9:**
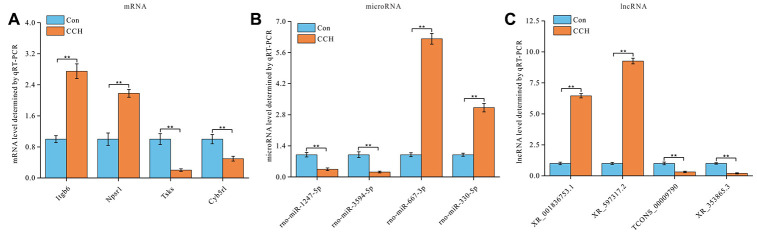
**Validation of lncRNAs-miRNAs-mRNAs regulatory network.** Four lncRNAs-miRNAs-mRNAs regulatory pairs were selected for validation by qRT-PCR (n=3). (**A**) mRNA relative level, (**B**) microRNA relative level, and (**C**) lncRNA relative level were showed. **, *P*<0.01.

To validate the ceRNAs levels of lncRNA-miRNA-mRNA regulatory network, four random lncRNA-miRNA-mRNA target pairs, Itgb6-miR-1247-XR_001836753.1, Npsr1-miR-3594-XR_597317.2, Tsks-miR-667-3p-TCONS_00009790, Cyb5rl-miR-330-XR_353865.3, were selected for qRT-PCR. The results showed that Itgb6 and Npsr1 levels were significantly upregulated and Tsks and Cyb5rl levels were significantly downregulated in hippocampus of CCH animals than that of sham animals (*P*<0.01) (Figure 9A). As predicted by lncRNA-miRNA-mRNA regulatory network construction, microRNAs rno-miR-1247-5p and rno-miR-3594-5p were significantly down-regulated, and rno-miR-667-3p and rno-miR-330-5p were significantly upregulated in hippocampus of CCH animals than that of sham animals (*P*<0.01) (Figure 9B). Similarly, lncRNA XR_001836753.1 and XR_597317.2 were significantly upregulated, TCONS_00009790 and XR_353865.3 were significantly down-regulated in hippocampus of CCH animals than that of sham animals (*P*<0.01) (Figure 9C). The primers used in qRT-PCR are listed in Table 4.

**Table 4:** Primers designed for qRT-PCR validation of lncRNAs and mRNAs.

**RNAs**	**Forward primer**	**Reverse Primer**	**Product length(bp)**
Itgb6	5' GGGTCCCTGAATGGTCCAAG 3'	5' CTCAGAGGCAGCAGTACCAC 3'	85
Npsr1	5' TCATCAAGCAACAGCTCCC 3'	5' AGCTGGAAAGAATGCATGAGGT 3'	142
Tsks	5' CGGGCAGAGTAGATGGTGAG 3'	5' CCCGCTTCGTGGATCTCAT 3'	106
Cyb5rl	5' TTGACACACATGTTGCCAC 3'	5' TGTCAAGGTCATCAGGCCAC 3'	161
rno-miR-1247-5p	5' AACAAGACCCGTCCCGTTC 3'	5' GTCGTATCCAGTGCAGGGT 3'	72
rno-miR-3594-5p	5' AACAATCCCAGGGCAGAGC 3'	5' GTCGTATCCAGTGCAGGGT 3'	82
rno-miR-667-3p	5' AACAATTGACACCTGCCACC 3'	5' GTCGTATCCAGTGCAGGGT 3'	72
rno-miR-330-5p	5' AACGATATCTCTGGGCCTGTG 3'	5' GTCGTATCCAGTGCAGGGT 3'	73
XR_001836753.1	5' CATGGAGACTTCTTAACACTGAG 3'	5' CCTTCAAAGCTGTCTGGCTTC 3'	139
XR_597317.2	5' ACCCAGACGTCCTCTTCCT 3'	5' TATGTTGTCGTTGGAGCCGT 3'	104
TCONS_00009790	5' CGAATACCCTCCCAGCTTCC 3'	5' TGTTCTGGCAGATTCCCAGTC 3'	76
XR_353865.3	5' CACACCGTTCCAGGGATTG 3'	5' CCGGATCCCAGCTTTTGAGT 3'	83

### Enrichment analyses of the DE lncRNA-miRNA-DE mRNA network

**Figure 10:**
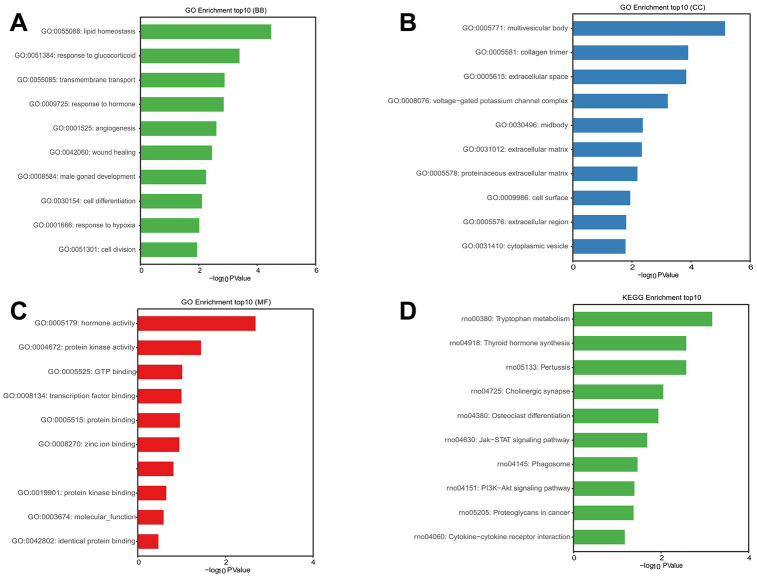
**GO and KEGG pathway enrichment analysis of mRNAs in ternary network of lncRNAs-miRNAs-mRNAs.** *P* value <0.05 was as significantly differentially enrichment. Enrichment value (-log10 (*P* value)) was calculated and visualized to show the enrichment of biological processes (**A**) the enrichment of cellular components (**B**) the enrichment of molecular functions (**C**) and the enrichment of the KEGG functional pathways (**D**).

To further predict and analyze the probable functions and pathway of DE mRNAs regulated by DE lncRNA-miRNA, GO and KEGG analysis were performed. The GO analysis showed that the most enriched mRNAs related to lipid homeostasis (GO: 0055088) in biological processes (Figure 10A), the multivesicular body (GO: 0005771) in the cellular component, and hormone activity (GO: 0005179) in the molecular functions (Figure 10B, 10C). KEGG pathway analysis showed that the top 10 deferentially-enriched KEGG pathways related to dysregulated mRNAs were involved in tryptophan metabolism, thyroid hormone synthesis, pertussis, cholinergic synapse, osteoclast differentiation, the Jak-STAT signaling pathway, phagosome, the PI3K-Akt signaling pathway, proteoglycans in cancer, and cytokine-cytokine receptor interaction (Figure 10D).

## DISCUSSION

Cognitive dysfunction caused by CCH is a common and important cause of many neurodegenerative diseases related to cognitive function. The mechanisms of cognitive dysfunction caused by CCH are not clear at present. LncRNA is a kind of noncoding RNA, which is believed to regulate gene transcription and expression. In this study, RNA sequencing analysis showed that 236 mRNAs and 189 lncRNAs were significantly altered in the CCH rat hippocampus when compared to sham controls. Among these, 128 mRNAs and 91 lncRNAs were up-regulated, and 108 mRNAs and 98 lncRNAs were down-regulated. This suggests that these changes in lncRNA and mRNA may function in the changes in brain structure, function, metabolism, and cognitive function caused by CCH.

Many RNA changes in this study are closely related to the changes in synaptic plasticity and cognitive function. Chat codes choline O-acetyltransferase (ChAT), which synthetizes acetylcholine, an important neurotransmitter of cholinergic neurons. Chat is closely related to cognitive function, and there are obvious changes in many stages of AD [26]. Ctnn codes cortactin, which fosters endo-lysosomal sorting and membrane surface distribution of AMPARs, and the LTP and LTD [27, 28]. Gabrr1 codes GABRR1, which is a GABA receptor that functions in neurotransmission of GABAergic neurons. Igf2 codes IGF2, which is one member of the insulin signaling pathway and its deficiency can lead to serious cognition dysfunction and it is involved in impaired cognition in Fragile X syndrome [29]. LOC108348065 codes histone deacetylase 6, which could balance and regulate cognition function. The genetic deletion of the histone deacetylase 6 exacerbates cognition deficits in the Huntington's disease mouse model [30], but reducing HDAC6 ameliorates cognitive deficits in an AD mouse model [31].

In order to better understand the biological functions and potential mechanisms of DE mRNA after chronic cerebral hypoperfusion, the GO and KEGG pathway enrichment analyses were conducted to explore this. The enriched terms in CCH consist of transporter activity (GO: 0005215), integrin binding (GO: 0005178), actin filament binding (GO: 0051015), adherens junction (GO: 0005912), the extracellular space (GO: 0005615), the monocarboxylic acid transport (GO: 0015718), and tryptophan metabolism, and so on. Most of these enriched terms are involved in synaptic plasticity, and myelin integrity, axonal transport and are related to cognition. Dopamine transporter (DAT) can enhance working memory [32]. The αvβ3 integrin receptor is involved in long-term potentiation and long-term depression [33]. The CAMs have been identified to increase synaptic strength at synapse, through recruiting scaffolding proteins, neurotransmitter receptors, and synaptic vesicles, which are closed with synaptic plasticity [34]. Rac1 enhances actin cytoskeleton by phosphorylating cofilin to remodel the structural spine and synaptic plasticity [35]. LncRNAs and their target miRNAs can competitively bind to mRNA as ceRNAs to up-regulate the level of protein-coding genes. Therefore, the lncRNA-miRNA-mRNA ternary network, via the Pearson correlation coefficient of the analysis of two pairs of lncRNAs, predicted miRNA and mRNA, was constructed to study the function of lncRNA regulating mRNA through miRNA. GO and KEGG pathway analyses for the mRNA in this ternary network were conducted to investigate the probable function and potential pathway regulated by lncRNA. The ternary network is involved the hormone activity (GO: 0005179), multivesicular body (GO: 0005771), lipid homeostasis (GO: 0055088), protein kinase binding (GO: 0019901), and transcription factor binding (GO: 0008134), and so on. Most of these enriched terms were also involved in cognition regulation. However, experiments are needed to verify how lncRNA regulates mRNA level through miRNA, and then participates in cognitive dysfunction after CCH. After all, the result inferred from bioinformatics is only a general idea.

LncRNAs can bind and regulate their anti-sense gene transcription in cis and be transported to other locations in the cell to regulate the transcription of genes by acting as scaffolds, decoys, guides, and enhancers to regulate mRNA transcription, and regulate post-translational mRNA level by sponging miRNAs in trans [19, 21]. In present study, we found 91 up-regulated lncRNAs and 98 down-regulated lncRNAs. Because of the numerous regulatory functions of lncRNA, lncRNAs whose levels changed in the hippocampus after chronic cerebral hypoperfusion were predicted to function in cognitive dysfunction. As there are many kinds of molecular up-regulation changes caused by stress response and compensation caused by chronic ischemia and hypoxia in the hippocampus of chronic cerebral hypoperfusion [36, 37], the up-regulated lncRNA may up-regulate the expression of protein molecules in stress and compensation after chronic cerebral hypoperfusion. At the same time, after chronic cerebral hypoperfusion, the energy synthesis of hippocampus decreased, and the synthesis of important protein molecules related to cognitive function decreased [38, 39]. The translation and synthesis of proteins are influenced by microRNA, enhancers, promoters and post-transcriptional modifications. LncRNA can function in these regulatory aspects. Therefore, the down-regulation of lncRNA may reduce the ability to protect mRNA from degradation and cut down mRNA level by decreasing the inhibition of lncRNA on microRNA. In addition, the down-regulation of lncRNA can reduce mRNA transcription by lessening the level of recruitment transcription factors and decreasing the signal guidance of transcription process. In both regulation methods, lncRNA and mRNA are co-expressed in neurons. By predicting the co-expression of lncRNA and mRNA, we can predict the possibility of some interaction or regulation between them. In the present study, we predicted and identified that 161 lncRNAs may be correlated with DE mRNAs via the Pearson correlation analysis. This suggests that these lncRNAs may be involved in the regulation of DE mRNA levels, but this needs to be verified by further experiments.

LncRNA has many functions involved in multiple known and unknown biological processes. The newly discovered neuron-specific nuclear lncRNA neuroLNC fostered presynaptic activity by interacting with TDP-43 [40]. As competitive endogenous RNAs, lncRNA and microRNA can function together to regulate the mRNA level in trans to prevent mRNA against degradation. In the present study, we identified 559 lncRNA-miRNA-mRNA target pairs, including 83 lncRNAs, 67 miRNAs, and 108 mRNAs. These potential triple pairs maybe take part in alterations of function, metabolisms, and structure after CCH, some of which could participate in cognition dysfunction. This potential functional network needs to be verified by further experiments. Such experiments may reveal some very important targets for improving cognitive impairment of CCH.

There were some limitations to our present study. The small sample size may have contributed to the improper estimation of DE RNAs. Future studies with larger sample sizes are needed to verify our present results. Our study results have not been experimentally validated and need verification by further experiments.

In conclusion, our results are helpful for understanding the pathogenesis of cognitive dysfunction caused by CCH and can provide direction for further research.

## MATERIAL AND METHODS

### Animals and CCH model surgery

Adult male Sprague-Dawley rats (200–220 g) were obtained from Hunan SJA Laboratory Animal Co., Ltd., and were housed with accessible food and water ad libitum. Rats were kept on a 12-h light/dark cycle with the light on from 7:00 am to 7:00 pm. Animal welfare and all experiments were approved by Ethics Committee of Renmin Hospital of Wuhan University.

The CCH model surgery was performed as previously described [12]. Briefly, the rats were anesthetized with intraperitoneal chloral hydrate (0.4 g/kg) and placed with a heating pad to maintain the body temperature at 37°C. After a ventral midline incision, both common carotid arteries were gently separated from the carotid sheath and vagus nerve [41]. Bilateral common carotid arteries were doubly ligated with 4-0 silk sutures just below the carotid bifurcation. In control rats, similar surgery was performed, but without vessel ligation. After the surgery was finished, the temperature of the rats was maintained under 37°C until recovery. During surgery, the brains of the rats did not soften, wholly or in part.

### Morris water maze

After 30 days of CCH, all rats completed spatial memory training in the Morris water maze. The experiment was conducted as previously described [39]. The rats were trained in the water maze to find a hidden platform. This training comprised four trials per day with a 30-s inter-trial interval between 2:00 and 8:00 pm for seven consecutive days. Each trial started with the rat placed in the middle of the outer edge of one quadrant and facing the wall of the pool, and ended when the animal climbed onto the platform. Rats that could not find the platform in 60 s were guided to the platform. The Morris water maze video tracking analysis system (Shanghai, China) was used to record the activity trajectory of the rats. The swimming paths of the rats and latencies of the rats to find the hidden platform were recorded [42]. The time the rat spent before arriving at the platform during the first trial on each day over a 7-day period was recorded as the latency time. Upon removal of the platform, which occurred during the fourth trial on each day over the 7-day period, the time the rats stayed in the platform area was recorded. The latency time and the number of times the rat crossed the platform area was used to evaluate learning ability. After 1 day of rest, the short-term memory retention test was performed. The platform was either present or absent and rats were put into the first quadrant of the maze. The latency time to reach the platform area, the number of times the rats crossed the platform area, and the total time spent in the platform quadrant, were recorded.

### HE staining

After finishing Morris water maze test, rats were anaesthetized by an overdose of chloral hydrate (1 g/kg), perfused, fixed and embedded with paraffins. Brains were cut into sections (5 μm) on slides. Paraffin section was dewaxed at 65°C after further dewaxing in xylene, the slices were hydrated in gradient alcohol. Then the sections were stained in hematoxylin staining solution for 5 minutes. After rinsing, the sections were separated color in alcohol hydrochloric acid for 45 seconds. After re-rinsing again, the sections were stained with eosin for 10 seconds and rinsed again, the slices were dehydrated with gradient alcohol and transparent in xylene. Finally, the sections were mounted with neutral gum and observed under microscope (Olympus BX51, Japan). The neuron morphology and number in hippocampus and striatum were observation and counted for analysis (number per mm^2^) to evaluate the neuronal pathological changes.

### RNA extraction and library preparation

Hippocampi tissue was separated from the rats’ brains, frozen in liquid nitrogen, and stored at -80°C until use. Total RNAs were extracted using the TRIzol reagent (Invitrogen, Singapore) according to the manufacturer’s protocol. RNA purity and quantification were evaluated using the NanoDrop 2000 spectrophotometer (Thermo Fisher Scientific, Waltham, MA, USA). RNA integrity was assessed using the Agilent 2100 Bioanalyzer (Agilent Technologies, Santa Clara, CA, USA). The qualified extracted hippocampal RNA samples must meet the following conditions: the sample concentration is not less than 100 μg/μL; total RNA quantity is greater than or equal to 1μg; the purity of samples requires OD260/280 value between 1.8-2.2; the sample integrity agilent2100 score is greater than or equal to 7 (RIN≥7). Then, the libraries were constructed using TruSeq Stranded Total RNA with Ribo-Zero Gold (Cat. No. RS-122-2301; Illumina, San Diego, CA, USA) according to the manufacturer’s instructions.

### RNA sequencing and differentially-expressed RNA (DE RNA) analysis

The libraries were sequenced on an Illumina HiSeq X Ten platform. After removing the adapter, ploy-N, low quality reads, and reads with length less 50 by Trimmomatic software, clean data (clean reads) were obtained. The original sequencing quantity, effective sequencing quantity, Q30 and GC content were counted and evaluated comprehensively. If the quality value is Q30, the probability of error recognition is 0.1%, that is, the accuracy rate is 99.9%. Qualified sequencing results require a quality value of at least 85%.

Sequencing reads were mapped to the human genome (GRCh38) using HISAT2 [43]. For mRNAs, the fragments per kilobase per million (FPKM) [44] of each gene was calculated using Cufflinks 2.0 [45], and the read counts of each gene were obtained by HTSeq-count [46]. Differential expression analysis was performed using the DESeq2 R package [47]. A *P* value < 0.05 was set as the threshold for significantly differential expression. For lncRNAs, the transcriptome from each dataset was assembled independently using the Cufflinks 2.0 program [45]. All transcriptomes were pooled and merged to generate a final transcriptome using Cuffmerge (Cufflinks 2.0). All transcripts that overlapped with known mRNAs, other non-coding RNA, and non-lncRNA were discarded. Next, the transcripts longer than 200 bp and with a number of exons > 2 were picked out, and CPC (v. 0.9-r2) [48], PLEK (v. 1.2) [49], CNCI (v. 1.0) [50], Pfam (v. 30) [51] were used to predict transcripts with coding potential. The novel predicted lncRNAs were obtained through these processes. The characteristics (including length, type, number of exons) of lncRNA were analyzed after screening. Then, the novel predicted lncRNAs and known lncRNAs (from the NCBI and Ensemble databases) were both used for expression calculation and differential screening. Then, differential expression analysis was performed using the DESeq (2012) R package. All sequencing process and analyses were performed using OE Biotech Co., Ltd. (Shanghai, China).

### Validation of quantitative real-time polymerase chain reaction (qRT-PCR)

To verify the validity and accuracy of RNA sequencing, we carried out qRT-PCR assessment to assess the data consistency between RNA sequencing and qRT-PCR. The RNA amplification by qRT-PCR has been described previously [12]. Total RNA of the 50 mg hippocampi tissue was extracted using the TRIzol Reagent according to the manufacturer’s protocol (Invitrogen). First strand complementary DNA (cDNA) was synthesized from total RNA using a First-strand cDNA Synthesis Kit (Thermo Fisher Scientific). The SYBR GREEN Mix (Invitrogen, Waltham, MA USA) reaction system was used for RT-PCR along with the forward primer, the reverse primer, and cDNA. The reaction process included: 1) a preincubation step at 95ºC for 3 min, 2) an amplification step of 45 cycles of 94ºC for 30 s, 3) different annealing temperature and 72ºC for 30 s, and 4) an elongation step of 72ºC for 10 min. A melting curve was recorded to verify the absence of primer dimers. Glyceraldehyde 3-phosphate dehydrogenase (GAPDH) was the endogenous control. RNA levels were assayed using the “ΔΔ Ct method” for relative expression.

### GO function and KEGG pathway annotation analyses

Gene Ontology (GO) annotated the gene function in three levels: molecular function, biological process, and cellular component. The main functions of the DE genes could be analyzed by comparisons with genes with annotated functions; therefore, the functions that may be enriched by DE genes were deduced through hypergeometric algorithms and tests to acquire the *P* value. Similarly, the probable pathway of DE genes was acquired by comparison with genes with an annotated pathway in the database of Kyoto Encyclopedia of Genes and Genomes (KEGG) pathways and by performing hypergeometric algorithms and other tests. A *P* value < 0.05 was considered statistically significant. The *P* value determined the degree of the function and pathway enrichment of the DE genes.

### LncRNA-mRNA co-expression analyses

For co-expression analysis of lncRNA and the gene, according to the expression levels of DE lncRNA and genes, the Pearson correlation analysis test was used to calculate the correlation between the two expressions. A pairing of a correlation coefficient of > 0.8 and a *P* value of < 0.05 was considered to indicate a co-expression relationship.

### ceRNA construction of the lncRNA-miRNA-mRNA network

The miRbase database and the miRanda program (v. 3.3a) [52] were used to predict the binding between these miRNA-differential mRNA/different lncRNA sequences, using the default parameters of miRanda v. 3.3a (S ≥ 150, ΔG ≤ −30 kcal/mol and demand strict 5' seed pairing), which predicts the miRNA bound to lncRNA and mRNA, respectively, and then takes the intersection to find the miRNA bound to both. S referred to the single residue pair match scores of the matching area, and ΔG referred to free energy of the double chains binding. Then, the intersecting miRNA and the corresponding lncRNA and mRNA were used to construct a ceRNA regulatory network of lnc-miRNA-mRNA using Cytoscape software [53].

### Statistical analysis

Data were expressed as means ± standard error of the mean (SEM) and analyzed using SPSS 20.0 statistical software (SPSS Inc., Chicago, IL, USA). The repeated-measures analysis of variance procedure was used to determine the statistical significance of RNAs among the three groups. The one-way analysis of variance procedure followed by Dunnett's t-test was used to determine the statistical significance of differences of the means for comparisons between the two groups. *P* < 0.05 was considered a statistically significant difference. Fold changes (FCs) and *P* value of tests were used to determine the statistical significance of the RNA sequence data. A FC ≥ 1.5 and *P* < 0.05 were used as thresholds for DE lncRNAs and mRNAs.
